# Philadelphia Academy of Surgery

**Published:** 1893-06

**Authors:** John B. Deaver


### PHILADELPHIA ACADEMY OF SURGERY.

MEETING MARCH 6TH, 1893.

THE PRESIDENT, DR. WILLIAM HUNT, IN THE CHAIR.

---

### AMPUTATION AT HIP-JOINT—ENCYSTED CARTILAGINOUS TUMOR NEAR SUB-CLAVIAN VESSELS—OPERATION ON THE FIFTH NERVE.

---

BY JOHN B. DEAVER, M. D.

*Mr. President and Fellows of the Academy :*

I will first present a case of amputation at the hip-joint, done for osteomyelitis of the femur. At the time of the operation the patient was very much depressed from sepsis, consequent upon prolonged suppuration. The only point of interest in the case from an operative point of view is that during the amputation hemorrhage was controlled simply by an Esmarch tube applied round the thigh, above the trochanter and along the crease of the groin, being retained here by two pieces of bandage, one passed beneath the tube in front and the other beneath the tube behind, each of which was held by an assistant. An oval flap of skin and facia was made, and the muscles divided down to the bone by a circular sweep of the knife. The superficial and deep femoral arteries, with their accompanying veins, were next tied separately, as well as those of the muscular branches which could be recognized. The tube was next loosened a little, and the small vessels, as they bled, caught with hæmostats. The tube was now removed, and an incision carried from the external angle of the wound up over the trochanter and into the joint dividing the capsular ligament, when the muscles were carefully separated from the bone and disarticulation completed. The amount of blood lost, I do not think, amounted to more than two ounces. The advantage this procedure offers over the Wyeth method is in not dividing the femur before the disarticulation is made, and further, that the amount of blood lost is not any greater, and that the vessels not being constricted for so long a time, there is less likelihood of consecutive bleeding. The tumor I here present is one of sarcoma, removed from the side of the neck, which had its origin from the periosteum of the vertebræ. The symptoms presented by the patient were those of laryngeal obstruction, paroxysmal in character and attended by the expectoration of large quantities of mucus. The symptoms of obstruction were not caused by pressure inflicted upon the larynx or trachea, but from involvement of the laryngeal nerves. Before the operation was performed I very much questioned if the removal of the growth would suffice to relieve the obstruction, which was afterward proven by the same symptoms continuing until death, twenty-four hours thereafter. The dissection was not a very difficult one, as the mass lay behind the large vessels, the pulsation of which was scarcely perceptible. The great amount of infiltration around the vessels must by necessity have involved the laryngeal nerves as well.

The second specimen is one of cyst, in the wall of which is a circular piece of cartilage. It was removed from the subclavian region of a man who was injured at the battle of Appomattox, April 9, 1865. When the accident occurred he was standing under a tree. He was not able to say, definitely, whether the injury resulted from being struck by a piece of shell or by a piece of wood from the tree. The only noticeable trouble at the time of the accident was fracture of the clavicle. From that time to the present a sinus has existed in the neck which patient states has been operated on without success. He was referred to me by Dr. Hildenbrand, when, upon examination, the orifice of the sinus was plainly to be seen, immediately above the inner end of the left clavicle, from which was escaping a purulent discharge and through which, upon the introduction of a probe, could be felt, what was believed, most probably, to be dead bone. Examination with the fingers demonstrated the presence of a partly movable mass which was thought to be a detached piece of the clavicle which had undergone necrosis. Operation revealed the presence of this cyst; it was attached to the sheath of the sutclavian vessels and to the pleura. Examination of the clavicle through the wound showed no trouble other than a slight enlargement at the seat of the original fracture. Examination of the cyst wall demonstrated very clearly the presence of cartilage.

T. M., aged fifty-eight years, white, Irish, slate-roofer; from a child had been very nervous, the slightest excitement or undue exertion throwing him into paroxysms of nervousness. When twenty-eight years of age, had an attack of smallpox which was followed by a weeping sore over the right inferior maxilla. This continued to discharge for six years, whcn it healed. Immediately after the healing of the sore he was attacked with neuralgic pains which were referred along the course of the inferior dental nerve. This pain continued at irregular intervals for six years, when he consulted a surgeon, who was supposed to have removed a section of the nerve near the mental foramen. Very little, if any relief followed this operation, when a second was performed by the same surgeon one year later; this was followed by relief for one year, when he had another attack of the pain. He now came under my care. I trephined the inferior maxilla over the angle and removed a section of the inferior dental nerve. This was followed by relief for a period of fifteen months, when the pain again returned. I now opened up the field of the old operation, exposed the proximal end (stump) of the nerve, excised a part therefrom, chiselled away the roof of the remaining portion of the dental canal, and removed the distal portion of the nerve as far as the mental foramen. This was followed by relief for sixteen months, when the pain returned, being referred, in addition to along the course of the inferior dental, along the side of the tongue. I now simply cleared out the field of the old operation, but this was not followed by any marked relief. I again operated, this time taking out a vertical section of the ramus of the jaw as far as the sigmoid cavity, and removed a further section from the proximal end of the inferior dental, and at the same time a section from the gustatory nerve. This was followed by relief. I purposely refrained from taking a section from the inferior maxillary nerve immediately after it passes through the foramen ovale, also from performing an intra-cranial operation, as I am not as yet, by any means, convinced that these more radical procedures are warrantable until the milder ones have been done without success. I can recall a number of cases, both of neuralgia of the inferior as well as of the superior maxillary nerve, where I have followed this course in relapsing attacks, with satisfactory results, to convince me that a longer period of relief from pain is offered the patient than would result, perhaps, by the more radical operations, removal of the Gasserian ganglion, etc., in the light of the present statistics.

DISCUSSION.

DR. JOHN B. ROBERTS: Twice I have had occasion to remove large malignant growth from the neck, and in both cases the result was the same as in Dr. Deaver's case. In one case, a child, I had to tie the internal carotid artery, and the child died on the seeond day with symptoms of brain implication. The other case was that of a man with a deep tumor requiring ligation, either of the internal jugular vein or of a large branch close up to the vein, I now forget which. I thought that he was going to get well, but he died on the fourth or fifth day with symptoms, the origin of which I could not determine. The wound was aseptic and nearly healed. He was found to be breathing very rapidly, and sank in a few hours in a sort of collapse. I could not tell whether there was implication of deeper organs or heart clot. No autopsy could be obtained.

DR. J. EWING MEARS: In the case which I reported and to which reference has been made by Dr. Deaver, I removed two and one-half inches of nerve and submitted it to Dr. DeSchweinitz for examination, and the condition found was that of fatty degeneration. It is important, it seems to me, that our studies should be directed toward ascertaining if possible, what the pathological condition is in these cases of trifacial neuralgia. I think that all of us have come to the conclusion that operative procedures appear in most cases, to be hopeless so far as permanent relief is concerned. It is impossible that from studies in regard to the cause of the condition, we may be able to indicate some method of operation which may prove more successful.

Last spring the members of the American Surgical Association were shown in the Massachusetts General Hospital the results in five or six cases of operations upon the second and third divisions of the fifth nerve for neuralgia. In these cases an incision had been made over the temporal region, the muscle cut through and the zygoma divided. By pressing the tissues down firmly the operator was able to reach the second and third divisions as they emerge from the foramen rotundum and ovale. In these cases the relief had extended, if I remember correctly, over three or four years, and in one case five or six years. From the reports which are given in Boston, this appears to be a very successful operation.

To my mind, the question of interest is in regard to the pathological condition. If the disease is of central origin I do not see how any operation on the peripheral terminations of the nerves can be of service. Repeated operations, such as Dr. Deaver performed, of course, give temporary relief.

DR. W. W. KEEN : I quite agree with Dr. Mears that the question of the pathology is a most important one. In the case where I have had a microscopical examination made the change has been found to be one of sclerosis. In one case there were spots of distinct hemorrhage into the nerve. These were almost macroscopic. I have never seen the inferior dental nerve so large as in this case. That patient had a return of the pain, and a second operation was done. So far as I could determine, a new nerve had formed, and, strange to say, there was a branch of this nerve which went inward through a foramen at the first operation. Dr. Dana some time ago published a paper in which he stated that he had found sclerosis of the vessels rather than of the nerve. However this may be, it seems to me clear that the sclerosis of the vessels or of the nerve is the chief thing, and that this is distinctly a senile change. That it does not appear in early life we all know, but only in later life, when sclerosis of other organs appear. This being the case, I think that the operation of choice should always be the peripheral operation. I should not think of endeavoring to remove or break up the Gasserian ganglion as a primary operation. I was told the other day that one of Mr. Rose's cases had shown symptoms of return, and this is what might be expected, as the sclerosis begins rather in the periphery and works backward. While medicine offers no benefit in the majority of cases, we can, as a rule, assure the patient that an operation will afford at least one or two years of relief. I presume that some of Dr. Deaver's operations consisted simply in reaming out the connective tissue about the stump of the nerve. This I have done in more than one case, and, although under the microscope no nervous tissue could be found in the material removed, the operation gave as much relief as followed a pure exsection of the nerve. This being the case, it seems to me that we should, as a general rule, endeavor to give relief by such a simple operation, rather than immediately to go to the foramen rotundum or ovale or within the skull and remove the Gasserian ganglion.

I notice that Dr. Deaver referred to destruction of the ganglion as not a serious operation. I should consider it quite a serious operation, although there have not been a large number of deaths. Rose has done it six or seven times, with one or two deaths. Andrews four times, without a death. Hartley once, with recovery, and Dr. Roberts once, with recovery. Besides this, two eyes, and possibly more, have been destroyed. It seems to me that any operation involving so much traumatism is to be considered a very serious operation, and should not be undertaken except after the gravest consideration.

DR. JAMES M. BARTON: As has been said by Dr. Mears, we have not yet arrived at the pathology of neuralgia. One suggestion is, that it is due to small aneurisms, which have been found in the diseased nerves. This view is supported by the results of the ligation of the external carotid for this affection.

Nussbaum claimed that one-half of the cases are permanently cured.

